# Alternative Inactivated Poliovirus Vaccines Adjuvanted with *Quillaja brasiliensis* or Quil-A Saponins Are Equally Effective in Inducing Specific Immune Responses

**DOI:** 10.1371/journal.pone.0105374

**Published:** 2014-08-22

**Authors:** Fernanda de Costa, Anna Carolina A. Yendo, Samuel P. Cibulski, Juliane D. Fleck, Paulo M. Roehe, Fernando R. Spilki, Grace Gosmann, Arthur G. Fett-Neto

**Affiliations:** 1 Plant Physiology Laboratory, Center for Biotechnology and Department of Botany, Federal University of Rio Grande do Sul (UFRGS), Porto Alegre, Brazil; 2 Faculty of Pharmacy, UFRGS, Porto Alegre, Brazil; 3 Fepagro Animal Health - Institute of Veterinary Research “Desidério Finamor” (IPVDF), Eldorado do Sul, Brazil, and Virology Laboratory, Microbiology Department, UFRGS, Porto Alegre, Brazil; 4 Molecular Microbiology Laboratory, Feevale University, Novo Hamburgo, Brazil; Federal University of São Paulo, Brazil

## Abstract

Inactivated polio vaccines (IPV) have an important role at the final stages of poliomyelitis eradication programs, reducing the risks associated with the use of attenuated polio vaccine (OPV). An affordable option to enhance vaccine immunogenicity and reduce costs of IPV may be the use of an effective and renewable adjuvant. In the present study, the adjuvant activity of aqueous extract (AE) and saponin fraction QB-90 from *Quillaja brasiliensis* using poliovirus antigen as model were analyzed and compared to a preparation adjuvanted with Quil-A, a well-known saponin-based commercial adjuvant. Experimental vaccines were prepared with viral antigen plus saline (control), Quil-A (50 µg), AE (400 µg) or QB-90 (50 µg). Sera from inoculated mice were collected at days 0, 28, 42 and 56 post-inoculation of the first dose of vaccine. Serum levels of specific IgG, IgG1 and IgG2a were significantly enhanced by AE, QB-90 and Quil-A compared to control group on day 56. The magnitude of enhancement was statistically equivalent for QB-90 and Quil-A. The cellular response was evaluated through DTH and analysis of IFN-γ and IL-2 mRNA levels using *in vitro* reestimulated splenocytes. Results indicated that AE and QB-90 were capable of stimulating the generation of Th1 cells against the administered antigen to the same extent as Quil-A. Mucosal immune response was enhanced by the vaccine adjuvanted with QB-90 as demonstrated by increases of specific IgA titers in bile, feces and vaginal washings, yielding comparable or higher titers than Quil-A. The results obtained indicate that saponins from *Q. brasiliensis* are potent adjuvants of specific cellular and humoral immune responses and represent a viable option to Quil-A.

## Introduction

More than 25 years after “The World Health Organization Polio Eradication Initiative” was established with the intention of eradicating poliomyelitis, a highly contagious disease that affects nerves and can lead to partial or full paralysis, remarkable success has been achieved in this field, with the reduction of global cases by 99% in 2013 [1]. Circulation of the virulent wild-type poliovirus strains has been eliminated in most countries and no cases of poliomyelitis caused by wild-type viruses have been reported in years [2]. Currently, however, virulent poliovirus strains continue to circulate in Nigeria, Pakistan, and Afghanistan [3]. Consequently, it is crucial to proceed with vaccination coverage worldwide, even in countries in which the virulent poliovirus strains no longer circulate, since the risk of poliovirus spreading from endemic to polio-free regions cannot be excluded [4], [5].

The use of inactivated polio vaccines (IPV) has an important role at the final stages of poliomyelitis eradication because it excludes the possibility of vaccine-associated paralytic poliomyelitis and vaccine-derived polioviruses, the two major drawbacks of the Sabin oral polio vaccine (OPV), a live attenuated vaccine. Nevertheless, the major obstacle to global IPV usage is that the cost per vaccine dose is too high, 5–15 times the current price of OPV, which makes it not an affordable option in several developing countries [4], [6]. One strategy to circumvent this problem is to reduce the antigen requirements per dose and, consequently, to lower costs of vaccine production. One of the ways by which this goal can be achieved is the use of viable adjuvants [6].

For about a decade, our research teams have been carrying out studies with *Quillaja brasiliensis* (Quillajaceae), a tree native of Southern Brazil. It is commonly known as “soap tree” in view of the folk use of its leaves as detergent, due to their high saponin content [7]. Chemical characterization of the saponins present in leaves of *Q. brasiliensis* and, particularly, of one saponin fraction, named QB-90, revealed compelling structural similarities with saponins from the barks of *Q. saponaria*, a related Chilean tree species and one of the main sources of industrial saponins - such as Quil-A - which has been widely studied and used as adjuvant in vaccine formulations [8]. In line with this observation, it has further been shown that aqueous extract (AE) and fraction QB-90 of the Brazilian species were able to stimulate both humoral and cellular immune responses against bovine herpesvirus types I (BoHV-1) and 5 (BoHV-5) in mice in a comparable manner to Quil-A saponins [9], [10].

An appropriate adjuvant, capable of inducing the adequate type of immune response to antigens, boosting the immune system for both humoral and cellular immune response, would be highly advantageous to the vaccine industry [11]. In addition, an adjuvant should have low toxicity and side effects, allowing it to be widely used in human or veterinary vaccine formulations. Quil-A use in vaccines has been restricted due to its reactogenicity, which includes an expressive hemolytic activity, local reactions and even systemic toxicity [12], [13]. Based on our previous work [9], [10] it was possible to conclude that *Q. brasiliensis* saponins presented significantly less *in vivo* and *in vitro* toxicity when compared to Quil-A, being considered a safer and just as effective alternative adjuvant.

The large scale use of *Q. saponaria* bark saponins has been compromising the sustainable production of this non wood-forest product. Because of the destructive effect of phloem stripping of trees during bark removal and the relatively slow growth of *Q. saponaria* forests, important ecological damage to Chilean forests has been reported [14] with a perspective of shortage of this resource to meet the vaccine industry demand. Consequently, the easily renewable use of bioactive saponins from *Q. brasiliensis* leaves assumes even more importance [15], [16]. In this work, we further advance knowledge on the adjuvant activity of saponins from leaves of *Q. brasiliensis* by analyzing the use of AE and QB-90 in an inactivated poliovirus vaccine, following immunization of mice. This study provides for the first time a direct comparison of the effect of *Q. brasiliensis* AE and QB-90 versus commercial Quil-A as vaccine adjuvants for triggering immune responses against a relevant human pathogen, including mucosal immunity, an important feature in polio vaccine.

## Materials and Methods

### Plant material and preparation of AE and saponin fraction QB-90

*Q. brasiliensis* leaves were collected from adult plants growing near Canguçu, RS, Brazil (31°23′42″S-52°40′32″W). A voucher specimen is deposited at the UFRGS Herbarium (ICN 142953). Air-dried powdered leaves were extracted in distilled water (1∶10, w/v) for 8 h, filtered, partitioned with ethyl acetate and lyophilized to obtain the AE. The AE was then submitted to further purification through reverse-phase chromatography to obtain fraction QB-90, as described in detail in previous work [9]. Quil-A was purchased from Brenntag Biosector (8047-15-2), Denmark.

### Poliovirus antigen preparation

For the preparation of poliovirus antigen, VERO cells were kindly provided by Dr. Clarice Weis Arns, from the Department of Genetics, Evolution and Bioagents (State University of Campinas, Brazil) and cultured at 37°C in a 5% CO_2_ incubator in Eagle's minimal essential medium (E-MEM; Gibco) supplemented with 10% fetal bovine serum and antibiotics (penicillin 100 IU/ml; streptomycin 100 µg/ml). Cells were subcultured every 3–4 days following standard procedures [17]. For virus multiplication, the poliovirus Sabin 1 strain was inoculated onto nearly confluent monolayers of VERO cells at a multiplicity of infection of 0.1. When cytopathic effect was evident in about 90% of monolayers, cells and supernatants were harvested and frozen at −80°C. Next, they were thawed, clarified by low speed centrifugation (1500×g for 10 min) and used as virus stocks. Titers obtained were around 10^7.5^ 50% tissue culture infectious doses per ml (TCID_50_) before inactivation with formaldehyde 0.1% (v/v) for one hour at 25°C [18]. The viral inactivation was confirmed by plaque assays of the 1∶10 and 1∶100 diluted suspensions in VERO cells and by three passages of the 1∶10 and 1∶100 diluted stocks on VERO cell monolayers.

### Mice and immunizations

Female Swiss mice of the CF-1 breed (60-days old) were purchased from the Fundação Estadual de Produção e Pesquisa em Saúde (FEPPS, Porto Alegre, RS, Brazil) and acclimatized for 72 h prior to use. Mice were maintained under controlled temperature (22±2°C) and humidity, under a cycle of 12/12 h day/night, and fed with standard pellet diet and tap water *ad libitum*.

Mice were divided into four groups, each consisting of seven mice. The formulations of poliovirus vaccines were filtered through 0.22 µm (Millipore) and kept at 4°C until use. Animals were immunized subcutaneously on the hind neck with 150 µl of poliovirus antigen plus 50 µl of one of the following adjuvants: QB-90 (50 µg), AE (400 µg), Quil-A (50 µg) or without adjuvant (antigen only), using saline as vehicle. A boosting injection was given 4 weeks later (day 28). Sera were collected on days 0, 28, 42 and 56 post-inoculation of the first dose of vaccine via tail vein and kept frozen until processed for determination of specific antibody titers in immunoassays. On day 56, four animals of each group were randomly selected and samples of different sites (fecal pellets, bile, and vaginal washing) were collected for assays in search for IgA. For fecal samples, three to four freshly produced pellets were collected from the distal colon, weighed, aliquoted and stored on ice. At the time of processing, ten volumes (per gram of wet weight of feces) of extraction buffer (10% fetal bovine serum, 0.01% sodium azide and 0.05% of penicillin/streptomycin/amphotericin B (100X) solution in PBS) were added to each aliquot. Tubes with such mixes were vigorously vortexed, centrifuged at 2,000×g for 20 min and the supernatant collected. Vaginal wash samples were collected by slowly injecting and withdrawing (3–4 times) 50 µl PBS (pH 7.2) into the vagina and the washing fluid volume recovered from each mouse was saved. To collect bile samples, the gall bladder was put into a micro tube and 125 µl of the extraction buffer were added. The gall bladder was macerated by cutting with scissors. The tube was centrifuged (10,000×g for 10 min), and the supernatant collected. All samples were stored at −20°C until assayed.

### Immunoassays

Anti-poliovirus IgG (total), IgG1 and IgG2a were quantitated in each serum sample by an indirect ELISA as previously described [9], [19]. ELISA plates (Nunc-Immuno MicroWell) were coated with the same poliovirus antigen used for mice immunization diluted as appropriate (1∶100, v/v) in carbonate-bicarbonate buffer pH 9.6 and incubated for 16 h at 4°C. After adsorption of the antigen, plates were washed once with 100 µl of PBS containing 0.05% Tween-20 (PBS-T), filled with 180 µl of PBS-T containing 2% casein and left standing for 30 min at 37°C. Subsequently, the plates were washed twice with PBS-T. Sera were appropriately diluted in PBS-T and added to wells in duplicate. After 1 h at 37°C, plates were washed three times with PBS-T and incubated with adequate dilutions of peroxidase conjugated anti-mouse IgG (Sigma), anti-mouse IgG1 (Sigma) or anti-mouse IgG2a (Sigma) for 1 h at 37°C. After washing, 100 µl of OPD (*ortho*-phenylenediamine) Sigma with 0.03% of H_2_O_2_ were added to each well. After 15 min of incubation in darkness at room temperature, the reaction was stopped with the addition of 1 M HCl (25 µl/well). Reading of the optical density was done in a microplate reader set to 492 nm. Data were expressed as the mean OD value of the samples minus the mean OD of control wells.

Anti-poliovirus IgA was determined in different mucosal sites in four animals of each group on samples collected on day 56 with the aid of an indirect ELISA, as follows. Aliquots (100 µl/well) of appropriately diluted samples (1∶1 for fecal samples, 1∶5 for vaginal washing and 1∶10 for bile samples) were added to duplicate wells of antigen-coated plates (see above), and plates were incubated at 4°C for 18 h. After washing three times with PBS-T, goat anti-mouse IgA (Sigma) (1∶4000) was added and incubated for 1 h at 37°C. After washing, anti-goat IgA (Sigma) (1∶5000) was added and plates again incubated for 1 h at 37°C. Color development with OPD and subsequent analyses were performed as described above.

### Delayed type hypersensitivity (DTH) assay

DTH responses were evaluated in three animals of each group on day 56. Mice were injected subcutaneously, in the right hind footpad, with 10 µl of poliovirus antigen used for immunization and the footpad thickness was measured with a calliper, both before and 24 h after injection. Injecting each animal with 10 µl saline in the left hind footpad served as controls. The poliovirus-specific response of each animal was calculated based on values of its injected footpad minus the average of the basal (control) swelling.

### Evaluation of cytokines gene expression

On day 56, four mice of each group had their spleens aseptically removed and disrupted in RPMI 1640 (Gibco) until homogeneous cell suspensions were obtained. Ammonium chloride (0.8%, w/v) was used for erythrocyte lysis and the cell suspension was centrifuged at 380×g for 10 min at 4°C. The pelleted cells were washed three times in RPMI and resuspended in RPMI 1640 medium supplemented with 10% fetal calf serum, 2 mM l-glutamine, 0.05 mM 2-mercaptoethanol and antibiotics (100 IU/ml of penicillin and 100 µg/ml of streptomycin). Splenocytes were counted by trypan blue exclusion and viability exceeded 99%. Cells were distributed in 96 well-microtiter plates (Nunc-Immuno MicroWell), each well seeded with 2.5×10^5^ cells in a final volume of 100 µl. Subsequently, poliovirus inactivated antigen diluted 1∶10 (100 µl) was added to a final volume of 200 µl, in triplicate. Cells were further incubated for a period of 16 h at 37°C under a 5% CO_2_ atmosphere and then stored at −80°C prior to use. RNA was extracted with PureLink RNA Mini Kit (Ambion), following manufacturer's instructions. Total RNA concentration was measured in UV spectrophotometer (Nanodrop, Thermo Scientific). First-strand cDNA synthesis was performed with Superscript III First-Strand (Invitrogen) reverse transcriptase using 1 µg of total RNA as template, 50 µM of Oligo d(T)_20_ primers, in presence of RNAse H (Life Technologies), according to the manufacturer's instructions.

The qPCR analyses were performed in fast optical 48-well reaction plates 0.1 ml (MicroAmp Applied Biosystems) using a StepOne Real-Time PCR System (Applied Biosystems), according to the manufacturer instructions. All of the reactions were carried out in triplicates for each cDNA sample and contained 1 µl 5-fold diluted cDNA template, 4 µl sterile water, 6.25 µl of Platinum SYBR Green qPCR SuperMix-UDG (Invitrogen), 0.25 µl of ROX reference dye, 0.5 µl of each of the 2 µM forward and reverse gene specific primers (see Table 1) in a final volume of 12.5 µl. Reactions were incubated at 95°C for 2 min to activate the Platinum Taq DNA polymerase, followed by 40 cycles of 95°C for 15 sec and 60°C for 30 sec. The specificity of the PCR was confirmed with melting curve from 60°C to 95°C, following the final PCR cycle. The expression levels of the genes of interest (interferon gamma, IFN-γ and interleukin 2, IL-2) were normalized using β-actin as reference gene [10]. The relative expression profile analysis was obtained through the 2^−ΔΔCt^ method [20], where ΔCt = Ct_gene of interest_ – Ct_β-actin_.

**Table 1:** Primer sequences for qRT-PCR amplification and product sizes.

Gene name	Primer sequence (5′-3′) forward/reverse	Amplicon length (bp)
*β-actin*	GCTTCTTTGCAGCTCCTTCGT/	68
	CGTCATCCATGGCGAACTG	
*IL-2*	CCTGAGCAGGATGGAGAATTACA/	92
	CTTTCAATTCTGTGGCCTGCTTGGG	
*IFN- γ*	TCAGCAACAGCAAGGCGAAA/	143
	CCGCTTCCTGAGGCTGGAT	

### Statistical analyses

Results were analyzed by ANOVA followed by Tukey test, whenever appropriate, using the statistic package SPSS 20.0 for Windows. Data were expressed as mean ± standard deviation (S.D.).

### Ethics statement

All experiments were performed in compliance with the European Convention for the Protection of Vertebrate Animals Used for Experimental and Other Scientific Purposes (European Treaty Series—No. 170 revised 2005) and the procedures of the Brazilian College of Laboratory Animals (COBEA). The experiments were approved by the Feevale University Committee for Animal Experiments (process 02.11.007).

## Results and Discussion

### Poliovirus-specific IgG and the subclasses in mice

**Figure 1:**
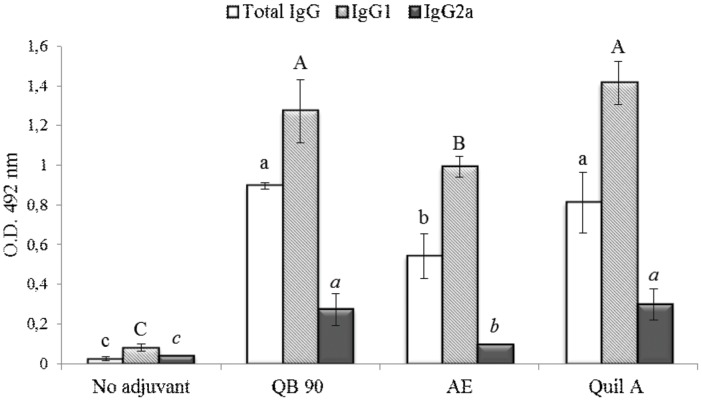
Serum titers of poliovirus total IgG, IgG1 and IgG2a isotypes. Sera were collected one month after the second antigen dose (day 56), administered either with no adjuvant or formulated with QB-90, AE or Quil-A. Results are expressed as the mean value ± S.D. (n = 7). Different lowercase, uppercase and italic letters indicate significant difference among the treatment samples of total IgG, IgG1 and IgG2a, respectively, by a Tukey test (ρ≤0.01).

Serum anti-poliovirus IgG, IgG1 and IgG2a were significantly higher in mice immunized with vaccine formulations containing QB-90 50 µg, AE 400 µg or Quil-A 50 µg, when compared to the control group (without adjuvant) on tests performed on samples collected on day 56 (**Fig. 1**). On day 42, total anti-poliovirus IgG, IgG1 and IgG2a profiles did not vary significantly in any of the samples (ρ≤0.05) (data not shown). Results of day 56 showed that, in all adjuvanted treatments, higher increases were observed for IgG and IgG1, compared to those detected for IgG2a. Vaccines formulated with QB-90 or Quil-A yielded statistically similar increases in anti-poliovirus IgG, IgG1 and IgG2a, whereas the AE formulation resulted in lower increases. However, the consistently significant yields of immunoglobulins induced by AE were quite impressive for a complex leaf aqueous extract.

In mouse, different Th1 and Th2 immune responses are divided in two distinct major subsets of T-cells, characterized by their pattern of cytokine production. Th1 responses are correlated with the induction of cell-mediated immunity and are usually associated with protection against intracellular pathogens, enhancement of IgG2a, IgG2b and IgG3 isotypes and production of cytokines IL-2, TNF-β and IFN-γ [21], [22]. On the other hand, Th2 responses are correlated with humoral immune response through the triggering of B cell proliferation and differentiation, with stimulation of production of IgG1 and the cytokines IL-4 and IL-10 [21].

In this context, the isotype patterns elicited by *Q. brasiliensis* saponins indicate that these, when used as adjuvants, are capable of inducing both humoral (Th2) responses, with induction of IgG1, and cellular (Th1) responses, with induction of IgG2a. The findings reported here provide further evidence in favor of the use of saponin extracts of this Brazilian species as adjuvants for vaccines against human pathogens, as initially indicated by studies with bovine herpesviruses [9], [10].

### DTH skin test and cytokine mRNA expression

**Figure 2:**
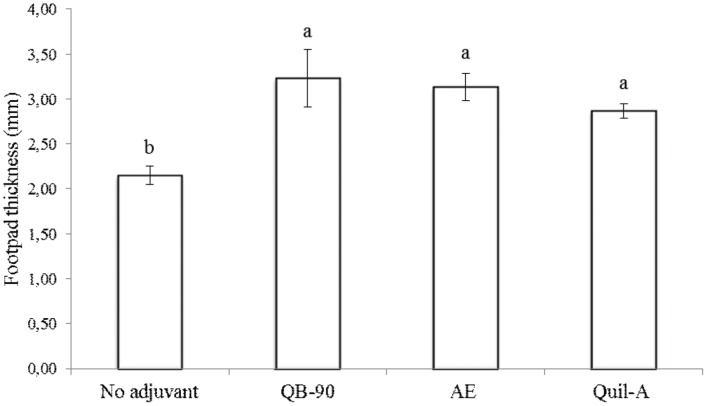
DTH reaction of immunized mice. The assay was carried out one month after the second immunization of mice with the poliovirus vaccine preparation (day 56) either not adjuvanted or with adjuvants QB-90, AE or Quil-A. The DTH response is expressed as the mean increase in thickness of the footpad in relation to controls (values ± S.D.); (n = 3). Different letters indicate significant difference by a Tukey test (ρ≤0.01).

Delayed-type hypersensitivity (DTH) is an *in vivo* manifestation of cell-mediated immune response, characterized by recruitment and activation of macrophages by CD4^+^ T-lymphocytes [23]. Macrophages initiate events through the presentation of antigens and production of inflammatory cytokines, such as IFN-γ and IL-2. The results of DTH assay (**Fig. 2**) revealed that animals immunized with QB-90- and AE-adjuvanted vaccines yielded significantly higher DTH responses than the control group. Besides, no significant differences in DTH responses were obtained between saponin extracts or fractions of *Q. brasiliensis* and Quil-A.

**Figure 3:**
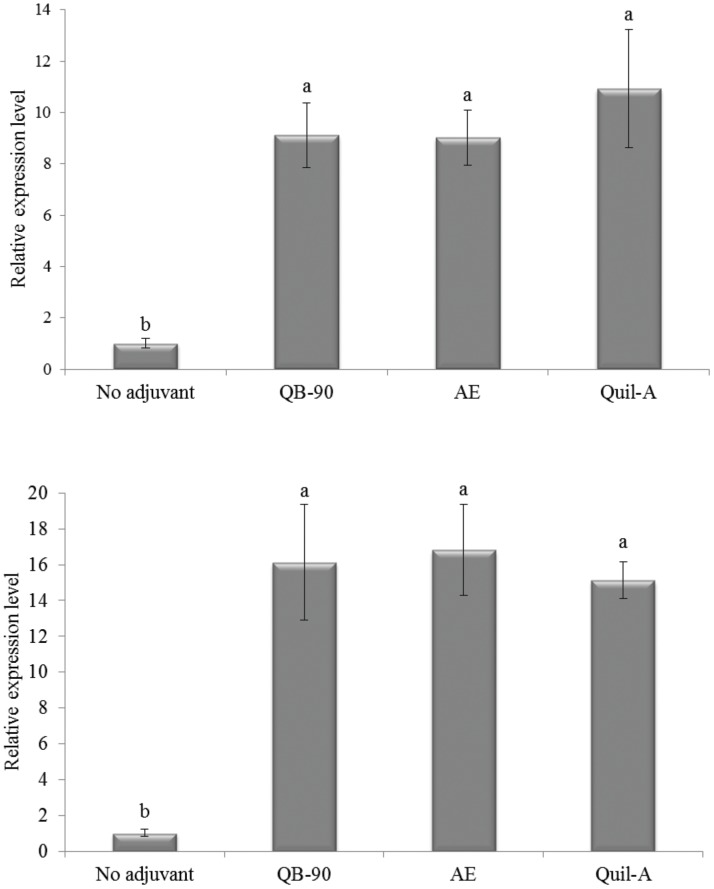
Relative expression profile of IFN-γ (a) and IL-2 (b) in poliovirus-reestimulated splenocytes from mice immunized with poliovirus vaccine containing either no adjuvant or formulated with QB-90, AE or Quil-A, one month after the second immunization. β-actin gene was used as reference to normalize mRNA expression and results were expressed as a ratio relative to the group that received non-adjuvanted vaccine, which was arbitrarily set to 1. The vertical bars represent the S.D. of four biological replicates. Different letters indicate significant difference by a Tukey test (ρ≤0.01).

Furthermore, mRNA expression levels of IL-2 and IFN-γ cytokines were evaluated by qPCR in antigen-stimulated splenocytes one month after the second immunization. As shown on **Fig. 3a**, QB-90- and AE-adjuvanted vaccines were able to induce transcripts of IFN-γ (almost tenfold) when compared to the non-adjuvant group. In addition, both adjuvants induced similar mRNA expression levels to those induced by the Quil-A-adjuvanted vaccine. Transcription of IL-2 (**Fig. 3b**) was strongly increased (almost 17-fold) by QB-90 and AE, followed by Quil-A (15-fold), when compared to no adjuvant group, despite the lack of statistically significant differences detected between vaccines prepared with either AE, QB-90 or Quil-A.

A significant increase of cytokines and a positive DTH reaction by QB-90 and Quil-A were shown when tested as adjuvants in vaccines prepared with bovine herpesvirus antigen [10]. Therefore, it is clear that *Q. brasiliensis* vaccine formulations resulted in stimulation of CD4^+^ T-cells and yielded a Th1 profile pattern of stimuli, characteristic of enhanced cellular immune responses.

### Elicitation of anti-poliovirus IgA responses at multiple mucosal sites

**Figure 4:**
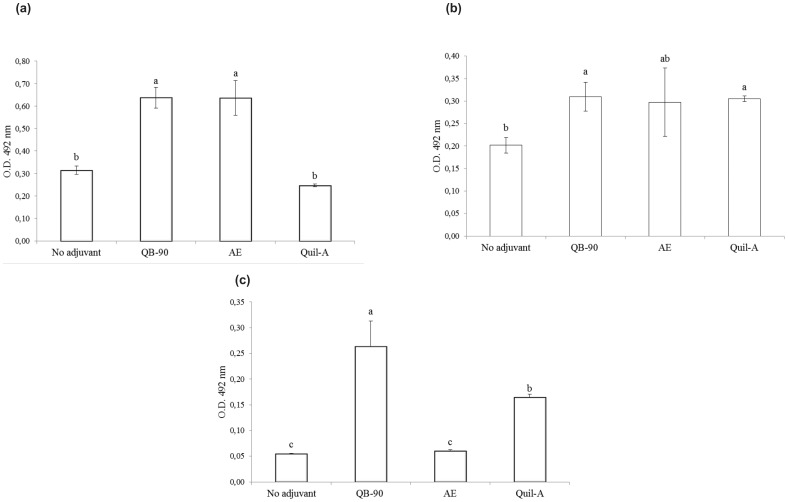
Poliovirus-specific IgA antibody responses in mice vaccinated with non-adjuvanted vaccine or QB-90-, AE-, or Quil-A- adjuvanted preparations in bile (a), fecal (b) and vaginal washings (c). Mice were euthanized at day 56 and bile, feces and vaginal washing samples were collected, processed and assayed for poliovirus-specific IgA antibodies. Results are expressed as the mean value ± S.D. (n = 4). Different letters indicate significant difference by a Tukey test (ρ≤0.05).

The potential of *Q. brasiliensis* adjuvanted vaccines to elicit mucosal immune responses upon subcutaneous immunization was evaluated one month after the second dose of vaccine (day 56). In bile samples, the groups immunized with QB-90- or AE-adjuvanted vaccines displayed significantly higher anti-poliovirus IgA antibody responses than the control group that was immunized with non-adjuvanted vaccine (**Fig. 4a**). Similar results were obtained for QB-90 in fecal and vaginal washings (**Fig. 4b, c**). In the group that received the AE-adjuvanted vaccine, a similar trend to increased levels of IgA was observed in fecal samples; however, no increases in IgA levels were detected in vaginal washing samples. When compared to Quil-A, vaccines with both QB-90 and AE displayed significantly enhanced levels of IgA in bile samples. In vaginal washings, the IgA concentration was significantly enhanced by the QB-90-adjuvanted vaccine.

Locally produced secretory IgA is the predominant immunoglobulin isotype along mucosal surfaces and is associated with reduced virus excretion after antigen challenge [24]. Most studies targeting mucosal immunity have used the native cholera toxin (CT) as adjuvant [25], [26]. CT has the advantage of being a reliable mucosal adjuvant; however, it is classified as a Th2-type adjuvant. Therefore, CT is not considered suitable for inducing systemic Th1-type cell-mediated immunity, which is important to combat intracellular infections, such as those caused by poliovirus. Saponins of *Q. saponaria* had already been targeted in studies of intranasal DNA vaccinations [27] and oral vaccinations [28], [29], showing increase of mucosal immune response with enhancement of IgA titers. To date, this is the first work that shows the potential of *Q. brasiliensis* saponins in achieving a mucosal immune response.

## Conclusions

Aiming at advancing the studies of *Q. brasiliensis* adjuvant activity and amplifying the range of antigens tested, a vaccine against an important infectious disease was evaluated in mice. Results herein described demonstrate that saponin formulations were able to enhance poliovirus- specific total IgG, IgG1 and IgG2a in systemic fashion, suggesting stimulation of both Th2 and Th1 immune responses. Cellular response stimulation was confirmed by DTH assays and by evaluating the enhancement of IL-2 and IFN-γ cytokine mRNA expression in mice splenocytes. Mucosal immune responses demonstrated that the QB-90-adjuvanted vaccine enhanced IgA titers in bile, feces and vaginal washings. The data gathered in this investigation, based on a vaccine against a clinically relevant virus for humans - though investigated in a murine model – provides additional evidence to support the use of *Q. brasiliensis* saponins as highly potent adjuvants, thus being a viable option to Quil-A. *Q. brasiliensis* saponins should be considered for further studies as an adjuvant that could make IPV more affordable, particularly in countries that are at center stage in the global efforts towards polio eradication.
